# Spiked Virus Level Needed To Correctly Assess Enteric Virus Recovery in Water Matrices

**DOI:** 10.1128/AEM.00111-19

**Published:** 2019-05-30

**Authors:** Qiaozhi Li, Yuanyuan Qiu, Xiao L. Pang, Nicholas J. Ashbolt

**Affiliations:** a School of Public Health, University of Alberta, Edmonton, AB, Canada; b Alberta Provincial Laboratory for Public Health, Edmonton, AB, Canada; c Department of Laboratory Medicine and Pathology, University of Alberta, Edmonton, AB, Canada; Rutgers, The State University of New Jersey

**Keywords:** QMRA, enteric viruses, recovery, spiking, water reuse

## Abstract

Quantitative microbial risk assessment (QMRA) identifies human enteric viruses in municipal wastewater as the pathogen group requiring the highest log reductions for various reuse applications. However, the performance of methods for estimating virus concentration is not well understood, and without performance assessment, actual risks are likely severely underestimated. To evaluate the efficiency of virus recovery from water, a water sample is often spiked with “known” amounts of virus, and the virus is then recovered after a series of analytical procedures. Yet for water matrices such as wastewater, due to the unknown background concentrations of targeted viruses in the matrix and the variable recovery efficiency between individual processes, only an approximation of the recovery efficiency may be obtained from such spike-and-recovery experiments. In this study, we demonstrated theoretically that for two widely used approximations, the error in estimating virus recovery should be less than the ratio of the amount of target virus in the background sample to that in the spike. Furthermore, we developed an applicable method, based on this new understanding, for deciding on the amount of virus for spiking before conducting a spike-and-recovery experiment, so that the approximation error is restricted to an acceptable level for each individual process. Finally, we applied the method to a set of experimental data for viruses in wastewater, demonstrating its utility and noting its general applicability to other pathogens or water matrices.

**IMPORTANCE** The performance of procedures for pathogen log reduction is at the heart of new risk-based guidance/regulation globally, yet the methods for undertaking assessments of pathogen recovery are not standardized despite their fundamental impacts on assessing log reductions. Here we describe the level of spiking agent(s) that is necessary to correctly assess spiked pathogen/surrogate recovery with whatever method is deployed. The significance of our research lies in identifying the importance of the amount of spiking agents for reducing uncertainty in recovery estimates, which will allow the development of a recommendation for spiking experiments, proactively applying this understanding.

## Abstract (precis)

The performance of procedures for pathogen log reduction is at the heart of new risk-based guidance/regulation globally, yet the methods for undertaking assessments of pathogen recovery are not standardized despite their fundamental impacts on assessing log reductions. Here we describe the level of spiking agent(s) that is necessary to correctly assess spiked pathogen/surrogate recovery with whatever method is deployed. The significance of our research lies in identifying the importance of the amount of spiking agents for reducing uncertainty in recovery estimates, which will allow the development of a recommendation for spiking experiments, proactively applying this understanding.

## INTRODUCTION

National standards for microbial hazards in harvested rainwater/storm water (1), as well as guidelines for direct potable reuse (2) and nonpotable reuse (3) of municipal wastewater recently accepted in the State of California (4), require specified log reductions in the amounts of enteric viruses, bacteria, and parasitic protozoa. There are significant uncertainties, however, when one is estimating microbial counts, due to matrix effects and associated variable losses with the different processing and data analysis methods used (5–9). Enteric virus estimation often involves more steps than for any other microbial group (10) and the highest log reductions for safe reuse (11). For example, analytical procedures for estimating infectious enteric virus concentrations may involve sample filtration, elution, concentration, culture, and quantitative PCR (qPCR) (10), and each step is known to cause inevitable loss of target viruses (9). The fraction of targeted virus in a water sample that is captured after the procedures is termed recovery efficiency or, more commonly, method recovery. Quantitative evaluation of method recovery is required to correct virus enumeration data for the fraction that is lost when one is using health-based, quantitative microbial risk assessment (QMRA)-derived log reduction targets (11–13).

When it is deemed necessary to use a spiked virus (14), known amounts of virions or viral genomic copies are added to a water sample, and recovery is then estimated by the difference in counts before and after processing of the water. The selection of spike material for a target virus has been discussed previously in the context of evaluating pathogen elimination in water treatment systems (14). In summary, lab-grown pure virus cultures are the least desirable, while viruses isolated from matrix water and propagated *in vitro*, or concentrated from matrix water, are preferred, because they are more likely to provide genetically and physiologically diverse populations that are representative of indigenous environmental strains (14). For the evaluation of method recovery as well, it is preferred that the spike material closely resemble the genetic and physiological variability of the indigenous target(s).

To avoid matrix effect complications, a control, target-free water sample is often included for spiking and measurement of method recovery. Under these circumstances, the method recovery for the target virus can be easily evaluated as the ratio of recovered to spiked viruses. However, water matrix effects are also important to address, since the physiochemical (e.g., temperature, pH, nutrients, and sorption to solids) and biological (predation by free-living protozoa and metazoa and enzymatic decomposition) factors of matrix water affect the amount of the target microorganism remaining in the water (15, 16). Thus, for greater relevance for performance-based log reduction assessments, matrix water is typically spiked with target microbes (14). Hence, our aim is to discuss, not the choice of spiked microbe, but rather how to evaluate the recovery of the target group with which the water matrix of interest has been spiked.

The main concern here is that the matrix water may also bring with it the target group of interest, which introduces an unknown and variable quantity into the recovery calculation. Furthermore, the variability in recovery efficiency between individual water processes makes it difficult to assess the recovered background target viruses by processing duplicated samples. Together, these factors make the calculation of recovery uncertain.

While spiking is not a new concept for method recovery related to enteric viral, bacterial, and protozoan pathogens and their surrogates (14), there is still no standard for how much spike control is needed. High titers of the selected spike material must be included in order to provide concentrations that are high enough to enable the evaluation of method recovery; however, it is unclear whether elevated or reduced recovery may result from too high a spiking dose. Therefore, the aims of this paper were to describe an optimum spike dose that is enough for assessing microbial method recoveries without risking artifacts in recovery estimates and to illustrate the approach with human enteric virus spiking of municipal wastewater.

## RESULTS

### Theoretical analysis.

**FIG 1:**
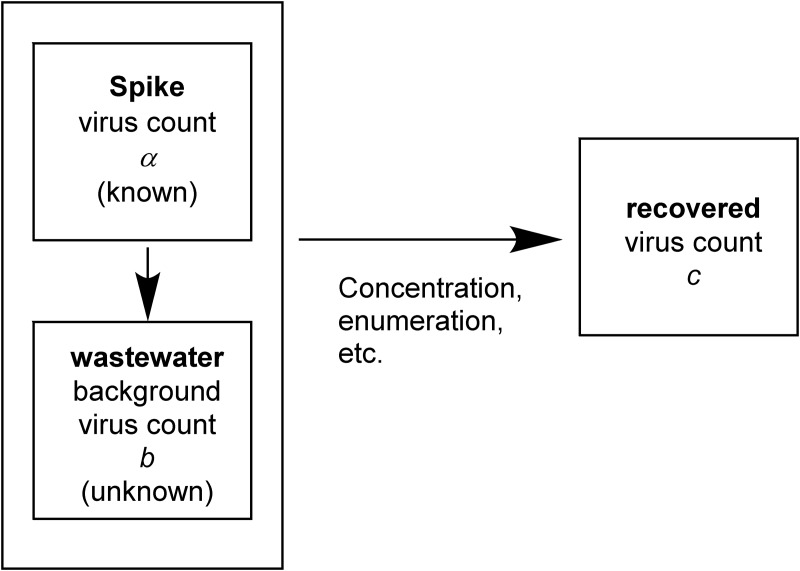
Diagram of a typical spike-and-recovery experiment for the evaluation of virus recovery in a water matrix.

Regardless of the spike material, it is imperative that the target in the spike be quantified (the virus count is quantified by the same method—either a molecular, culture, or mixed method—throughout the experiment) prior to inoculation. The target spike is then mixed with related microbes present in the matrix water sample, and the target is (partially) recovered with a certain recovery efficiency. A typical spike-and-recovery experiment for viruses is diagramed in Fig. 1.

By definition, the overall recovery efficiency (*r*) is expressed as follows:

(1) $$r=\frac{c}{a+b}\left(0\leq r\leq 1\right)$$

where *a* is the virus count in the spike, *b* is the indigenous virus count in the matrix water, and *c* is the virus count that is recovered and quantified.

Since the virus count in the matrix water sample (*b*) is unknown, it is challengeable to calculate the exact recovery efficiency from an individual sample, but approximations can be obtained using the following two widely used approaches.

### (i) Approximation I.

When the virus count in a matrix water sample (*b*) (e.g., wastewater, surface water, etc.) is very low compared to the virus count in the spike (*a*), i.e., *b*/*a* ≈ 0, it follows that

(2) $$\overset{˜}{r}=\frac{c}{a}$$

Then $\overset{˜}{r}$ in equation 2 is a good approximation of the recovery efficiency *r* in equation 1. In practice, the virus count in the spike (*a*) is controllable. In order to keep the condition for equation 2 (i.e., *b*/*a* ≈ 0) valid, we are able to adjust the spike dose (*a*) based on the background virus count in the matrix water sample (*b*). Furthermore, we need to understand how *b*/*a* relates to the error of approximation of recovery efficiency $\overset{˜}{r}-r$ so that we will be able to determine how close *b*/*a* should be to zero in order to keep the error in the approximated recovery efficiency acceptable (for example, <1%, <2.5, or <5%), a level that is chosen subjectively.

To discuss the error of approximation I, let

$$k=\frac{a}{b}\left(b>0\right)$$

Then the error of the approximated recovery efficiency can be expressed as

$$\overset{˜}{r}-r=\frac{\mathrm{cb}}{a\left(a+b\right)}=\frac{b}{a}\frac{c}{a+b}=\frac{r}{k}$$

Since 0 ≤ *r* ≤ 1, it follows that

$$0\leq \overset{˜}{r}-r\leq \frac{1}{k}$$

so recovery can be described in equation 3 as follows:

(3) $$\overset{˜}{r}-\frac{1}{k}\leq r\leq \overset{˜}{r}$$

Therefore, using $\overset{˜}{r}$ to approximate recovery efficiency *r*, the true recovery efficiency r will be overestimated, and the maximum error is less than 1/*k*.

### (ii) Approximation II.

When there is no variation presented in recovery efficiencies across individual samples, a duplicate sample collected at the same time could be used jointly to estimate the mean recovery. Suppose that *d* virus particles are recovered from the duplicate sample without spiking, which has the same background virus count b as the spiked sample, it follows that the recovery efficiency for the duplicate sample without spiking (*r*′) is

(4) $${r\prime }^{}=\frac{d}{b}$$

and its value should be the same as r defined for the sample with spiking in equation 1, i.e., *r*′ = *r*. Combining equation 1 and equation 4, we could get equation 5.

(5) $$r=r\prime =\frac{c-d}{a}$$

However, it is known that virus recovery can differ considerably across samples (9); thus, the condition for equation 5, i.e., *r*′ = *r*, is unlikely to be valid under most circumstances. For general circumstances, taking

$$\overset{˜}{r}\prime =\frac{c-d}{a}$$

as an approximation of the recovery efficiency r in equation 1 will introduce an approximation error. We will discuss how *b*/*a* relates to the error of approximation of recovery efficiency $\overset{˜}{r}\prime -r$ as follows in approximation II.

For approximation II, following the above approach:

$$\overset{˜}{r}\prime -r=\frac{\left(a+b\right)(c-d)-\mathrm{ac}}{a(a+b)}$$

$$=\frac{\mathrm{bc}}{a\left(a+b\right)}-\frac{d}{a}$$

$$=\frac{b}{a}r-\frac{b}{a}r\prime$$

$$=\frac{r-r\prime }{k}$$

It follows that

$$\left|\overset{˜}{r}\prime -r\right|=\frac{\left|r\prime -r\right|}{k}\leq \frac{1}{k}$$

Since 0 ≤ *r* ≤ 1 and $0\leq \overset{˜}{r}\prime \leq 1$, we derive equation 6, which demonstrates that the error of approximation II is between –1/*k* and 1/*k*.

(6) $$\overset{˜}{r}\prime -\frac{1}{k}\leq r\leq \overset{˜}{r}\prime +\frac{1}{k}$$

**FIG 2:**
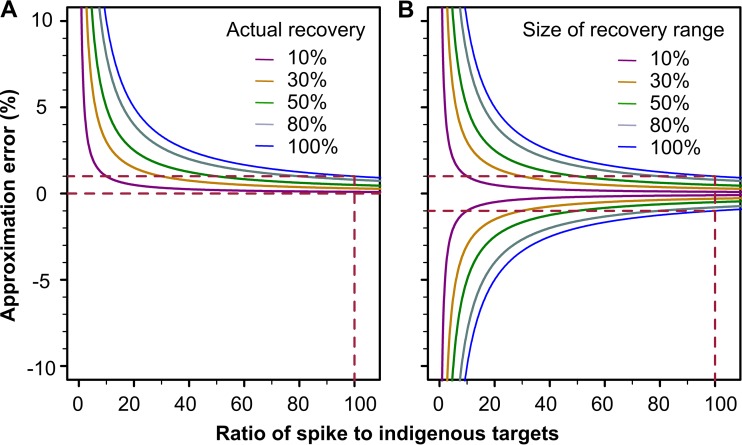
Approximation error in relation to the ratio of the microbial target count in the spike to that in the background sample. (A) Approximation I; (B) approximation II.

The relationship between the approximation error and the ratio of the target (virus) count in the spike to that in the background matrix water sample is presented in Fig. 2.

For a matrix water sample with unknown true recovery efficiency, using either approximation I or approximation II, the approximation errors in the worst-case scenarios are represented by blue lines in Fig. 2. When the ratio of the microbial target count in the spike to that in the background water sample (*k*) is large, the approximation error can be neglected for either approximation I or approximation II. Based on this understanding, for a predetermined error level σ (for example, 5%), one can spike the matrix water sample with an appropriate microbial target (e.g., virus) count so that the maximum error term 1/*k* is less than σ, or *k* > 1/σ, for which approximations I and II both provide acceptable approximations of the true recovery efficiency.

To keep *k* > 1/σ so that the error in the approximated recovery efficiency is less than the predetermined error level σ, it is required that

(7) $$a>\frac{b}{\sigma }$$

In practice, however, the spike-in microbial target count (*a*) cannot be determined directly based on the choice of *k*, because the microbial target count in the background water sample (*b*) is unknown. Some extra information needs to be collected prior to the spike-and-recovery experiment so that the background virus count *b* can be evaluated approximately. Specifically, the following procedure is recommended in order to choose the spike-in microbial target count (*a*).

(i) Collect *n* replicate samples (a minimum of two replicates is recommended considering the difference in the background virus count expected between replicates), and then process the water in the same way as the test sample, except without a spike step. It is assumed that the replicates have the same background target concentration. We term the maximum recovered target amount *d*^0^, and we term its corresponding unknown background target count and unknown recovery efficiency *b*^0^ and *r*^0^, respectively. When *n* is large, it is most likely that *d*^0^ corresponds to the sample with both the largest background target count and the highest recovery efficiency considering the variation of replicate samples and the variation of recoveries.

(ii) Obtain the lowest recovery efficiency observed for a cleaner water matrix as an approximation for the recovery efficiency (for example, virus recovery values obtained from spike-and-recovery experiments for pure water). It is likely that the recovery for pure water is higher than that for other water matrices, so we treat the minimum recovery efficiency for pure water as an approximation of *r* in equation 1.

Since the replicate samples have the same background target concentration, *b*^0^ = *d*^0^/*r*^0^ is the most likely higher and closest approximation of *b*, based on the available information.

It is worth noting that the aim here is to obtain the best spiking dose *a*. Since *b*/σ is the lowest that *a* can go to ensure that the recovery estimation is valid (equation 7), choosing a larger approximation of *b* in the calculation would result in a larger spiking dose than the lowest value of *a* but would still fulfill the requirement. It is also the lowest value of *a* that is above its unknown lowest value and a value we are confident in working with. Therefore, the minimum dose of the microbial target count required for spike-in is approximated as *d*^0^/σ *r*^0^.

### Application of spiking approaches to estimation of the recovery of human enteric viruses.

The spike-and-recovery requirement of the minimum value of *a*/*d*^0^ to deliver an approximation error of 5% for approximations I and II (equations 3 and 5, respectively) is shown in Table 1. For the human enteric virus data presented in Table 1, all samples analyzed were above the limit of detection (LOD) of 1 virus/sample volume, except for reovirus in unspiked samples (24/25 and 13/14 samples for wastewater treatment plant 1 [WTP1] and WTP2 were below the LOD, respectively, and half the LOD was substituted.)

**TABLE 1:** Spike-in requirement for the Calgary enteric virus wastewater study^*a*^

Virus	*r*^0^ (%)^*a*^	Minimum of *a*/*d*^0^ for an error of <5%	No. of samples excluded/total no.		Mean (SD) recovery results (%)			
					Approximation I		Approximation II	
			WTP1	WTP2	WTP1	WTP2	WTP1	WTP2
Norovirus GII	32	62.6	22/32	21/21	10.4 (6.0)	ND^*b*^	10.2 (5.9)	ND
Adenovirus	20	100	4/32	9/21	13.7 (14.4)	11.5 (5.4)	13.5 (14.5)	11.2 (5.4)
Enterovirus	32	62.6	2/32	13/21	21.5 (6.6)	31.9 (4.8)	21.0 (6.6)	31.1 (4.9)
Reovirus	1^*c*^	2,000	0/25	0/14	18.9 (15.5)	19.1 (11.8)	18.9 (15.5)	19.0 (11.8)

a Minimum reference recovery (17); data from reference 18.

b ND, no data.

c Since no data were available, 1% was assumed.

## DISCUSSION

As expected, when the results in Table 1 are compared with those presented in a previous publication (17), the wastewater matrix has a lower virus recovery efficiency in general than that obtained by spiking pure water (for norovirus GII, 32% to 69%; for adenovirus 41, 20% to 52%; and for an enterovirus [coxsackievirus B], 32% to 69%). Another important consideration is the LOD for viruses, which is relevant to the interpretation of nondetects. When the virus concentration in the wastewater is low, i.e., when the virus count in the matrix water sample (*d*) might be under the LOD, the *a*/*d*^0^ ratio could be calculated using the LOD as a conservative estimate of *d*^0^.

Although approximations I and II both gave acceptable approximations of recovery efficiency when the spike-in virus count fulfilled the condition for equation 5, we can choose between approximations I and II if we have more information about the distribution range of the recovery efficiency. For example, for viruses, we could assume that the recovery efficiency itself is highly variable (9); hence, approximation I would be preferable to approximation II. However, when the recovery efficiency itself is large but spans only a small range of values, it is better to choose approximation II over approximation I.

In practice, it is desirable to determine recovery with each water sample; however, this might be costly and laborious, depending on the detection method. When it is impractical to have a replicate for every sample (for example, in monitoring programs with time series of samples), it would be acceptable to collect duplicate samples only at selected times with high virus loadings for the same water source, assuming that some information on likely times of higher virus loadings is available. The highest recovered background virus count among all duplicate samples should then be used (as *d*^0^) for calculating the preferred spiking dose. This simple approach will result only in a higher value of the spiking dose and is not likely to alter the estimation for recovery efficiency.

We discussed the appropriate spiking dose for spike-and-recovery experiments, focusing on viruses. But the principles are generally true for method recovery for any pathogen or microbial surrogate, and for all other water matrices, such as river water, storm water, etc.

## MATERIALS AND METHODS

The methods described in this paper for determining the target microbe count in the spike were applied to a set of human enteric virus recovery data for municipal wastewater treatment plant (WTP) performance. In brief, wastewater samples subjected to secondary treatment were collected in duplicate at the pre-UV disinfection site from two WTPs in Calgary, Canada (18). A spiking mix consisting of four viruses (norovirus GII, human adenovirus 2/4, reovirus 2, and an enterovirus, coxsackievirus B [derived from human stool, human stool, wastewater, and cell culture, respectively]), was used to assess virus recoveries. One of the duplicate samples was spiked with 1 ml of the virus mixture. Then all samples were processed similarly by concentration, viral nucleic acid extraction, and qPCR to estimate total virus concentrations. Finally, the same aliquot of the virus mixture (1 ml) was added to 14 ml of deionized water as a baseline control, which was tested in parallel with the concentrated spiked water samples using qPCR. A detailed description of sample collection and processing has been given previously (18).

The spike-in virus count was determined based on estimation of the count in pure water; therefore, we are not sure whether the spike-in virus count is enough for a reliable approximation of the recovery efficiency based on equation 5, which could be expressed as follows:

(8) $$\frac{a}{d^{0}}>\frac{1}{\sigma r^{0}}$$

The minimum requirement for *a*/*d*^0^ to deliver an approximation error of 5% was calculated based on equation 8. Then for each set of duplicate samples, we calculate the ratio *a*^0^/*d*^0^, where *a*^0^ is the qPCR results of the spike and *d*^0^ is the qPCR results of the duplicate sample without spiking. As a result, duplicate samples that did not have an *a*^0^/*d*^0^ ratio above the threshold value were excluded from the recovery efficiency calculation.
